# Assessing the risk of sarcopenia among community-dwelling older adults in Israel: a national cross-sectional survey

**DOI:** 10.1007/s41999-025-01297-7

**Published:** 2025-09-15

**Authors:** Miri Lutski, Ziv Karni-Efrati, Inbar Zucker, Dvora Frankenthal

**Affiliations:** 1 The Israel Center for Disease Control (ICDC), Ministry of Health, Sheba Medical Center, Ramat Gan, Israel; 2 School of Public Health, Gray Faculty of Medical and Health Sciences, Tel Aviv University, Tel Aviv, Israel; 3 Center for Research and Study of Aging, University of Haifa, Haifa, Israel; 4 Central Department of Health, Ministry of Health Israel, Jerusalem, Israel

**Keywords:** Israel, Older adults, Risk assessment, Risk for sarcopenia, Screening, Survey

## Abstract

**Purpose:**

With aging population and the growing burden of sarcopenia on health systems, early risk assessment is essential. This study assessed the risk of sarcopenia and associated factors among community-dwelling older adults.

**Methods:**

This cross-sectional study used data from the Israel National Elderly Falls Survey (2018–2019) among adults aged 65+ by the Israel Center for Disease Control. Data were collected via telephone interviews on demographic, health, and functional characteristics. Sarcopenia risk was assessed using the validated five-item Mini Sarcopenia Risk Assessment (MSRA-5) questionnaire, with a cutoff score of ≤ 45 indicating risk. Multivariable logistic regression estimated odds ratios (ORs) and 95% confidence intervals (CIs) for sarcopenia risk.

**Results:**

A total of 2668 participants were included in the study, with a mean age of 73.2 ± 5.7 years, and 56.3% were female. Based on the MSRA-5 cutoff score (≤ 45), 65.1% (*n* = 1738) were classified as at risk for sarcopenia. Older age (OR = 1.21 per 5-year increase, 95% CI 1.10–1.33), Arab ethnicity (OR = 2.05, 95% CI 1.59–2.64), lower education level (OR = 1.57, 95% CI 1.29–1.92), and anxiety/depression (OR = 1.83, 95% CI 1.32–2.54) were associated with the prevalence of sarcopenia risk. Functional limitations, including difficulty performing household activities (OR = 1.96, 95% CI 1.42–2.69) and physical inactivity (OR = 1.72, 95% CI 1.40–2.11), were also associated with sarcopenia risk. A dose–response relationship was observed with medication use, with progressively higher odds of sarcopenia risk among those taking > 8 medications per day (OR = 2.75, 95% CI 1.52–4.98).

**Conclusion:**

These findings highlight the high prevalence of sarcopenia risk among older adults in Israel and emphasize the importance of early screening to identify at-risk individuals.

## Key summary points

**Aim:**

The aim is to assess the risk of sarcopenia and associated factors among community-dwelling older adults in Israel.

**Findings:**

Based on a nationally representative survey, 65.1% of adults aged 65 and older were at risk for sarcopenia and identify key associated factors including older age, Arab ethnicity, lower education, polypharmacy, physical inactivity, and functional limitations.

**Message:**

Early screening for sarcopenia should be integrated into routine care for older adults, particularly those with known risk factors.

## Introduction

Sarcopenia, a progressive and generalized skeletal muscle disorder, is characterized by the loss of muscle mass, strength, and function with aging [1]. The term, derived from the Greek words meaning “poverty of flesh,” was first introduced by Rosenberg in 1989 to describe the age-related decline in muscle mass [2]. Sarcopenia is now recognized as a major public health concern due to its strong association with frailty, disability, falls, hospitalization, and mortality in older adults [1].

The aging population in Israel is growing rapidly; recent demographic data indicate that individuals aged 65 and older constitute approximately 12% of the total population and this proportion is expected to increase significantly in coming decades [3]. This demographic shift highlights the urgent need to address age-related conditions such as sarcopenia, which can substantially impact independence and healthcare utilization among older adults.

Sarcopenia can be classified into primary and secondary types based on its underlying causes. Primary sarcopenia occurs in older adults without identifiable causes beyond the natural aging process. In contrast, secondary sarcopenia develops when additional factors beyond aging lead to reductions in muscle mass as well as impairments in muscle strength and physical function. These factors include chronic diseases (particularly inflammatory conditions), prolonged inactivity, disability, and nutritional deficiencies due to low protein or energy intake [1, 4].

The prevalence of sarcopenia varies widely depending on the population studied and the diagnostic criteria applied. Although sarcopenia is increasingly recognized for its clinical significance, a single universally accepted definition has not been adopted globally. A systematic review and meta-analysis by Petermann-Rocha et al. highlighted high variability in sarcopenia prevalence estimates, largely due to differences in diagnostic criteria and cut-off values [4]. The revised European Working Group on Sarcopenia in Older People (EWGSOP2) criteria are most commonly used in Europe [1] while in the United States, the Sarcopenia Definitions and Outcomes Consortium (SDOC) has proposed alternative diagnostic criteria that place greater emphasis on functional outcomes [5]. In Asia, the Asian Working Group for Sarcopenia (AWGS) criteria are widely used [6]. Recently, the Global Leadership Initiative on Sarcopenia (GLIS) has proposed a consensus conceptual framework aimed at harmonizing these diverse definitions to improve consistency in research and clinical practice across countries [7–9]. These developments reflect a growing global effort to standardize the assessment of muscle mass, strength, and function in aging populations.

A recent systematic review and meta-analysis of 263 studies reported sarcopenia prevalence ranging from 8 to 36% in individuals under 60 and 10–27% in those 60 and older [4]. Severe sarcopenia ranged from 2 to 9%. Prevalence varied by sex and criteria used: it was higher in men using EWGSOP2 (11 vs. 2%) and in women using the International Working Group criteria (17 vs. 12%) [4]. Validated screening tools are essential to detect sarcopenia early and enable timely intervention. While imaging techniques provide precise muscle mass evaluation, they are costly and not feasible for routine screening. Practical tools like the Mini Sarcopenia Risk Assessment (MSRA) and SARC-F offer quick assessments. While SARC-F relies on subjective self-reporting, the MSRA is based on objective and measurable parameters such as age, physical activity, recent hospitalization, weight loss, and dietary habits [10]. The MSRA exists in two formats—MSRA-7 and MSRA-5—with the latter being more concise and practical for large surveys. Studies have shown that the MSRA-5 offers higher sensitivity than SARC-F but somewhat lower specificity [10]. Rossi et al. demonstrated that an MSRA-5 cutoff of 45 yields a sensitivity of 80.4% and a specificity of 60.4%, with performance comparable to the MSRA-7 [11]. Given its brevity, ease of use, and strong screening performance, we selected the MSRA-5 as a first-line tool suitable for community-dwelling older adults.

In Israel, data on sarcopenia are limited and mostly focus on specific subgroups [12–14]. To date, no national estimate of sarcopenia risk prevalence has been conducted. Given the growing aging population and the significant burden sarcopenia puts on health systems, assessing its risk among community-dwelling older adults is critical for early intervention and prevention strategies. Therefore, this study aimed to assess the risk of sarcopenia and identify factors associated with it among community-dwelling adults aged 65 and older who participated in the national cross-sectional Israel National Elderly Falls (INEF) Survey.

## Methods

### Study design

This cross-sectional study is based on data obtained from the first representative Israel National Elderly Falls (INEF) Survey conducted by the Israeli Center for Disease Control. This survey was part of a national program initiated by the Israeli Ministry of Health (MOH) to reduce the incidence of falls in the older population [15]. The survey was conducted between February 2018 and April 2019 and included 3181 Israeli community-dwelling individuals aged 65 and above. A detailed description of the survey design and procedures has been published previously [15]. In brief, a national random sample of telephone numbers (mobile and landline) of Jewish and Arab households was extracted from a national database that encompasses over 90% of households with at least one telephone line, whether landline or mobile. This sample was selected using an algorithm that accounts for the geographic distribution of population groups (Jewish and Arab) in Israel, ensuring that each district is appropriately represented based on the relative size of each population group in that area. Households without telephone access and those with no residents aged 65 or older were excluded from the survey. Trained personnel conducted telephone interviews, collecting data on demographic characteristics, comorbidity, functioning, health-related characteristics, and anthropometric measurements.

According to Israeli legislation, telephone health surveys are conducted under the regulatory authority of the Israel Center for Disease Control (Ministry of Health) and do not require approval from an ethics committee. All participants provided verbal informed consent following a brief explanation of the survey’s purpose and importance. Consent was documented in the questionnaire, and all collected data remained anonymous. For the current study participants who did not respond to all five questions included in the five-item Mini Sarcopenia Risk Assessment questionnaire (MSRA-5) were excluded (*n* = 513, 16.1%). The age and sex distribution of the final sample was representative of the older adult population in Israel. Additionally, we compared the distribution of key variables—age, sex, population group, and education—between the full Israel National Elderly Falls Survey sample and the analytic sample. The comparison revealed no significant differences in the distribution of these variables.

### Sarcopenia risk assessment

Sarcopenia risk was assessed using the MSRA-5, a validated tool designed for community-dwelling adults aged 65 and older [11, 16]. The questionnaire includes five questions: (1) age (< 70/≥ 70), (2) hospitalization in the past year (yes, more than once/yes, once/no), (3) activity level (able to walk < 1000 m/≥ 1000 m), (4) meal frequency (skipping meals up to twice a week/eating three meals daily), and (5) unintentional weight loss in the past year (> 2 kg/≤ 2 kg). As the MSRA-5 had not been previously validated in Israel, we translated the original English version into Hebrew and performed a back-translation into English by two independent translators to ensure consistency. The Hebrew version was pretested among older adults to confirm comprehension, and no cultural or contextual adaptations were deemed necessary. A dichotomous measure was applied using an MSRA-5 cutoff score of ≤ 45, where scores at or below this threshold indicate a risk for sarcopenia [11].

### Socio-demographic and health-related variables

Data on education (≤ 12 years/> 12 years), sex, and population group (Jews/Arabs) were collected through self-reports provided by the participants.

Comorbidities were assessed by asking participants, “Has a doctor ever diagnosed you with any of the following chronic diseases: chronic heart failure, diabetes, arthritis, anxiety and/or depression, osteoporosis?” (yes/no). Medication use was evaluated with the question, “How many different types of medications (excluding vitamins, minerals, and nutritional supplements) do you take per day?” Responses were categorized into four groups: no medications, 1–4 medications, 5–8 medications, and more than 8 medications per day. Polypharmacy was defined as the use of ≥ 5 medications daily. Body mass index (BMI) was calculated based on self-reported weight in kilograms (measured with light clothing and no shoes) divided by the square of height in meters (measured without shoes). BMI was then classified into the following categories: underweight (< 18.5 kg/m^2^), normal weight (18.5–24.9 kg/m^2^), overweight (25.0–29.9 kg/m^2^), and obese (≥ 30 kg/m^2^). Functional status was evaluated by asking, "Do you have any difficulty performing household activities such as cleaning or shopping?" The response options included: no difficulty, slight difficulty, great difficulty, or inability to perform these activities. For the purposes of this assessment, experiencing difficulty in performing household activities was considered as “yes” if the responses indicated either great difficulty or an inability to perform them. Responses of “no difficulty” or “slight difficulty” were categorized as “no”. Physical activity was assessed by asking, “In your free time, do you usually engage in exercise to maintain fitness and health?” (yes/no).

### Statistical analysis

Sociodemographic and health-related characteristics of the participants were expressed as frequencies and percentages for categorical variables and as the mean and standard deviation for continuous variables. We performed univariate analyses including Chi-square and *t*-tests to compare participants’ characteristics according to sarcopenia risk groups (MSRA-5 ≤ 45 vs. > 45).

Multivariable logistic regression was used to estimate odds ratios (ORs) and 95% confidence intervals (CIs) for sarcopenia risk (MSRA-5 ≤ 45 vs. > 45), using the enter method. Potential interactions were assessed between all covariates. Socio-demographic variables, including age, sex, education level, and population group, were included in the model regardless of statistical significance in the univariate analysis. Health-related variables (i.e., comorbidities, BMI, polypharmacy, functional status, and physical activity) were included only if they demonstrated a significance level of *p* < 0.1 in univariate analyses. Odds ratios (ORs) with 95% confidence intervals (CIs) were calculated to estimate the strength of associations, with statistical significance set at *p* < 0.05. Data were analyzed using SPSS version 29.0 (SPSS, Chicago, IL).

## Results

A total of 2668 participants were included in the study. The mean age of participants was 73.2 ± 5.7 (mean ± SD) years, and more than half were females (56.3%). Patients’ distribution according to the Mini Sarcopenia Risk Assessment 5 items questionnaire is detailed in Table 1.

**Table 1:** Patients’ distribution according to the Mini Sarcopenia Risk Assessment 5 items questionnaire

Items	Score	All sample
		*N* = 2668
		*N* (%)
How old are you?		
≥ 70 years	0	1961 (73.5)
< 70 years	5	707 (26.5)
Where you hospitalized in the last year?		
Yes, and more than one hospitalization	0	226 (8.5)
Yes, one hospitalization	10	335 (12.6)
No	15	2107 (78.9)
What is your activity level?		
I'm able to walk less than 1000 m	0	899 (33.7)
I'm able to walk more than 1000 m	15	1770 (66.3)
Do you eat 3 meals per day regularly?		
No, up to twice per week I skip a meal	0	1051 (39.4)
Yes	15	1617 (60.6)
Did you lose weight in the last year?		
> 2 kg	0	643 (24.1)
≤ 2 kg	10	2025 (75.9)

Around one-fifth of participants reported at least one hospitalization in the past year. Nearly one-third of participants were unable to walk more than 1000 m, and about 40% skipped a meal up to twice per week. Additionally, almost 25% of participants experienced a weight loss of at least 2 kg over the past year. Based on the accepted MSRA ≤ 45 cutoff point, 65.1% of participants were classified as at risk for sarcopenia. This included 64.6% (*n* = 753) of men and 65.5% (*n* = 985) of women. The socio-demographic and health-related characteristics of the participants are detailed in Table 2. Compared to participants in the not-at-risk group (*n* = 930, 34.9%), those at risk for sarcopenia (*n* = 1738, 65.1%) were older and had significantly higher proportions of Arabs, individuals with ≤ 12 years of education, and those with obesity, chronic heart failure, diabetes, anxiety or depression, arthritis, or osteoporosis. Additionally, a higher proportion of participants in the risk group for sarcopenia reported consuming ≥ 5 medications per day. Both groups had a similar distribution of sex. We assessed interactions and potential multicollinearity between variables and found them insignificant.

**Table 2:** Socio-demographic and health-related characteristics according to Sarcopenia Risk Assessment groups (MSRA-5 ≤ 45 vs. > 45)

Characteristics	All sample	MSRA-5 ≤ 45	MSRA-5 > 45	*p* value
	*N* = 2668	*N* = 1738 (65.1%)	*N* = 930 (34.9%)	
Age, years (mean ± SD)	73.2 (± 5.7)	73.9 (± 5.9)	72.0 (± 5.1)	< 0.001
Sex, *N* (%)				0.61
Male	1165 (43.7)	753 (43.3)	412 (44.3)	
Female	1503 (56.3)	985 (56.7)	518 (55.7)	
Population group, *N* (%)				< 0.001
Jews	1809 (67.8)	1032 (59.4)	777 (83.5)	
Arabs	859 (32.2)	706 (40.6)	153 (16.5)	
Years of education, years, *N* (%)				< 0.001
≤ 12	1400 (52.5)	1069 (61.5)	331 (35.6)	
> 12	1268 (47.5)	669 (38.5)	599 (64.4)	
Chronic heart failure, *N* (%)				< 0.001
Yes	160 (6.2)	133 (8.0)	27 (2.9)	
No	2416 (93.8)	1527 (92.0)	889 (97.1)	
Diabetes, *N* (%)				< 0.001
Yes	881 (33.3)	633 (36.7)	248 (26.9)	
No	1767 (61.7)	1093 (63.3)	674 (73.1)	
Anxiety/depression, *N* (%)				< 0.001
Yes	370 (14.0)	303 (17.7)	67 (7.3)	
No	2268 (86.0)	1413 (82.3)	855 (92.7)	
Arthritis, *N* (%)				< 0.001
Yes	446 (17.20)	375 (22.2)	71 (7.8)	
No	2150 (82.8)	1311 (77.8)	839 (92.2)	
Osteoporosis, *N* (%)				0.04
Yes	509 (20.0)	351 (21.2)	158 (17.8)	
No	2039 (80.0)	1307 (78.8)	732 (82.2)	
Body mass index, *N* (%)				< 0.001
Underweight (> 18.5)	20 (0.8)	14 (0.9)	6 (0.70)	
Normal weight (18.5–24.9)	721 (29.7)	416 (26.8)	305 (34.9)	
Overweight (25–29.9)	1085 (44.7)	689 (44.4)	396 (45.3)	
Obese (≥ 30)	602 (24.8)	434 (27.9)	168 (19.2)	
Medication use, *N* (%)				< 0.001
0	315 (11.8)	162 (9.3)	153 (16.5)	
1–4	1564 (58.6)	961 (55.3)	603 (64.8)	
5–8	582 (21.8)	433 (24.9)	149 (16.0)	
> 8	207 (7.8)	182 (10.5)	25 (2.7)	
Difficulty to perform household activities, *N* (%)				< 0.001
Yes	2106 (80.5)	1256 (74.0)	850 (92.5)	
No	511 (19.5)	442 (26.0)	69 (7.5)	
Physical activity, *N* (%)				< 0.001
Yes	1400 (52.6)	756 (43.7)	644 (69.2)	
No	1261 (47.4)	975 (56.3)	286 (30.8)	

MSRA-5: Mini Sarcopenia Risk Assessment score; Missing: chronic heart failure = 92, Diabetes = 20, Anxiety/depression = 30, Arthritis = 72; Osteoporosis = 120, Body mass index = 240, Difficulty to perform; household activities = 51, Physical activity = 7

Multivariate analysis identified several significant factors for sarcopenia risk (Table 3). Age, analyzed in 5-year intervals, demonstrated a significant association with sarcopenia risk (OR = 1.21, 95% CI 1.10–1.33). Being Arab (OR = 2.05, 95% CI 1.59–2.64, *p* < 0.001), having ≤ 12 years of education (OR = 1.57, 95% CI 1.29–1.92), and the presence of anxiety/depression (OR = 1.83, 95% CI 1.32–2.54) were also associated with an increased sarcopenia risk. Physical functionality indicators also showed significant associations, with difficulty performing household activities (OR = 1.96, 95% CI 1.42–2.69) and physical inactivity (OR = 1.72, 95% CI 1.40–2.11) being notable risk factors.

**Table 3:** Adjusted odds ratio and 95% confidence interval for sarcopenia risk (MSRA-5 ≤ 45) [multivariable logistic regression]

Variables^a^	OR	CI 95%	*p* value
Gender			
Male	1 (ref)		
Female	1.07	0.88–1.31	0.47
Age (in 5-year intervals)	1.21	1.10–1.33	< 0.001
Population group			
Jew	1 (ref)		
Arab	2.05	1.59–2.64	< 0.001
Years of education			
> 12	1 (ref)		
≤ 12	1.57	1.29–1.92	< 0.001
BMI			
Normal weight (18.5–24.9)	1 (ref)		
Overweight (25–29.9)	1.16	0.96–1.48	0.19
Obese (≥ 30)	1.32	1.001–1.74	0.05
Anxiety/depression			
No	1 (ref)		
Yes	1.83	1.32–2.54	< 0.001
Chronic heart failure			
No	1 (ref)		
Yes	1.63	1.004–2.66	0.04
Arthritis			
No	1 (ref)		
Yes	1.54	1.12–2.12	0.007
Number of medications^b^			
0	1 (ref)		
1–4	1.34	1.001–1.80	0.05
5–8	1.56	1.07–2.28	0.02
> 8	2.75	1.52–4.98	< 0.001
Difficulty in performing household activities			
No	1 (ref)		
Yes	1.96	1.42–2.69	< 0.001
Physical activity			
Yes	1 (ref)		
No	1.72	1.40–2.11	< 0.001

*OR* odds ratio, *BMI* body mass index, *MSRA-5* Mini Sarcopenia Risk Assessment score

^a^The following variables diabetes, and osteoporosis were entered into the regression model but were found to be not statistically associated with a risk of sarcopenia

^b^A Cochran–Armitage linear trend test was also performed to check a dose response relationship (*Z* = 10.19, *p* < 0.001)

A dose–response relationship was observed with medication use, where the risk of sarcopenia increased with the number of medications taken (Cochran–Armitage test for linear trend: *p* < 0.001) (Table 3). Compared to those taking no medications, participants taking 1–4 medications (OR = 1.34, 95% CI 1.001–1.80), 5–8 medications (OR = 1.56, 95% CI 1.07–2.28), and > 8 medications (OR = 2.75, 95% CI 1.52–4.98) showed progressively higher odds of sarcopenia risk.

Notably, diabetes and osteoporosis, while included in the regression model, did not show statistically significant associations with sarcopenia risk.

## Discussion

The findings of this study provide valuable insights into the prevalence and risk factors associated with sarcopenia risk in a nationwide survey of community-dwelling older adults aged 65 and older in Israel. To our knowledge, this is the first national study in Israel to assess sarcopenia risk prevalence. Using the MSRA-5 screening tool, we found that approximately two-thirds (65.1%) of the study population were classified as at risk for sarcopenia, with no significant difference observed between men and women.

This finding is consistent with a cross-sectional study that assessed sarcopenia risk using the Polish version of the MSRA-5 questionnaire among volunteers aged 60 and older, reporting a prevalence of 59.4%, with a slightly higher but non-significant rate among women [16]. Sarcopenia risk prevalence varies widely across studies due to differences in population characteristics, assessment tools, and diagnostic criteria. For example, a cross-sectional study from Belgium comparing five screening methods found that the estimated prevalence of sarcopenia ranged from 5.7 to 16.7% among adults aged 65 and older, depending on the method used and the population studied [4, 17]. Another cross-sectional study from Thailand evaluating the validity of MSRA-5 and other screening tools reported sarcopenia risk prevalence of 21.5, 72.3, and 61.5% when using the SARC-F, MSRA-7, and MSRA-5, respectively, among adults aged 60 and older attending outpatient clinics [18]

Our findings further emphasize the importance of sociodemographic factors, such as increasing age, lower educational attainment, and Arab ethnicity, all of which were significantly associated with a higher risk of sarcopenia. The association between lower education levels and sarcopenia risk aligns with large studies indicating that individuals with less education have a higher prevalence of sarcopenia [19, 20]

One possible explanation is that individuals with lower educational attainment may have limited health literacy, reduced access to health-promoting resources, and less awareness of nutritional and physical activity guidelines, all of which are key factors in preserving muscle mass and function in older age [20]. While previous Israeli studies reported no ethnic differences in handgrip strength [21] and even indicated greater frailty among Jewish and Arab populations [22], our results support international findings of ethnic disparities in sarcopenia risk. For example, in the United States, Du et al. [23] reported that sarcopenia and sarcopenic obesity prevalence varied significantly by race/ethnicity, with non-Hispanic Blacks and Hispanics showing higher rates compared to non-Hispanic Whites. In China, Liu et al. [24] found differences in sarcopenia prevalence between ethnic groups using the AWGS criteria, with higher prevalence observed among ethnic minorities relative to the Han majority. Similarly, in the Netherlands, the HELIUS study demonstrated that sarcopenia prevalence and protein intake differed across ethnic groups, with higher risk among Turkish, Moroccan, and Surinamese older adults compared to native Dutch participants [25]. These disparities are likely influenced by differences in physical activity, nutrition, socioeconomic status, and healthcare access. In our study, the elevated sarcopenia risk observed among Arab older adults may reflect broader socio-cultural and environmental factors within Israeli society [26]. Compared to Jewish older adults, Arabs may be more likely to experience certain socioeconomic challenges, have varying levels of access to healthcare, and engage in different patterns of physical activity [27, 28]. Other contributing factors could include differences in dietary habits, health literacy, and cultural perceptions related to health behaviors [26]. These complex and interrelated factors highlight the importance of developing culturally sensitive prevention strategies and call for further research to better understand the underlying mechanisms.

Our findings underscore the link between health-related factors, such as anxiety/depression, chronic heart failure, arthritis, and sarcopenia risk. The strong association with anxiety and depression may be explained by their impact on lifestyle behaviors; individuals with depression are more likely to engage in less physical activity and have poorer nutritional intake, both of which are critical for maintaining muscle mass and function. It is also important to consider that the relationship between anxiety/depression and sarcopenia may be bidirectional. Reduced muscle strength and physical limitations associated with sarcopenia can, in turn, lead to psychological distress, diminished self-efficacy, and social isolation, potentially exacerbating symptoms of depression and anxiety [29]. This is consistent with previous research showing that depressive symptoms, particularly in women, increase sarcopenia risk [30]. The connection between arthritis, anxiety, depression, and sarcopenia may also be driven by shared biological mechanisms, including oxidative stress and inflammation [31, 32]. In line with prior studies, we observed a higher risk of sarcopenia among individuals with rheumatoid arthritis, a condition associated with increased risks of falls, fractures, and disability, thereby emphasizing its clinical importance [32].

We found that a higher number of medications was associated with an increased risk of sarcopenia. This aligns with the Berlin Aging Study II, which showed that taking ≥ 5 medications daily was linked to increased exhaustion, reduced gait speed, and a higher likelihood of sarcopenia, even after adjusting for confounders [33]. Similarly, a Japanese longitudinal study found that older adults prescribed ≥ 6 medications daily had a greater risk of developing sarcopenia [34]. Possible explanations for this association include age-related changes in drug metabolism, increased exposure to medications that may contribute to muscle loss and impaired function, and the cumulative effects of multiple prescriptions, where one medication leads to additional prescriptions to manage side effects [35]. Another possible explanation is that the use of multiple medications is a marker of poorer overall health, which can lead to reduced physical activity and inadequate nutritional intake, both key risk factors for sarcopenia [33].

The study indicated that individuals reporting significant difficulty or inability to perform household activities were at a two-fold higher risk for sarcopenia, compared to those who did not encounter such difficulties. This finding aligns with a retrospective study that investigated the utility of another screening tool (SARC-F) using data from the African American Health Study, the Baltimore Longitudinal Study of Aging, and the National Health and Nutrition Examination Survey among the U.S. population. In that study, instrumental activities of daily living (IADL) deficits observed during follow-ups were associated with a SARC-F ≥ 4 score, indicating a higher risk of sarcopenia [36].

Our study found that physical inactivity was associated with an increased risk of sarcopenia. This aligns with a systematic review and meta-analysis showing that inactive older adults have a higher risk of sarcopenia [29]. Data from the CHARLS study further support this, indicating that engaging in vigorous physical activity for at least 30 min once a week reduces the risk of probable sarcopenia, defined in that study as low muscle strength (handgrip strength < 28 kg in men and < 18 kg in women) or poor physical performance (5-time chair stand test ≥ 12 s) [37]. It is worth noting that sarcopenic obesity may have been present in some CHARLS participants, potentially compounding the impact of low muscle strength and excess adiposity on physical function. Sarcopenic obesity, characterized by the coexistence of low muscle mass and elevated fat mass, is associated with inflammation, insulin resistance, and impaired mobility [38]. Physical inactivity can exacerbate this condition, creating a vicious cycle of muscle degradation and metabolic decline [39]. Therefore, prevention strategies should integrate nutritional support, clinical care, and structured physical activity programs tailored to older adults. The current findings show that participants with a higher BMI had an increased risk of sarcopenia, though the association was only borderline significant. This aligns with an Israeli cross-sectional study of men with cardiovascular disease, which linked overweight and obesity in midlife to late-life sarcopenia [12]. Aging leads to increased visceral fat and decreased muscle mass, with fat-induced inflammation contributing to sarcopenia [40, 41]. This cycle of muscle loss and fat gain can worsen inflammation and glucose intolerance [40]. However, these changes may be reversible through lifestyle modifications [41].

Recent research has focused on developing personalized strategies for the prevention and treatment of sarcopenia [42–45]. Early identification of individuals at risk for sarcopenia is crucial, as timely interventions can help prevent or slow disease progression. Being classified as "at risk" does not necessarily mean that an individual has sarcopenia, but rather that they exhibit characteristics such as physical inactivity, poor nutrition, older age, or weight loss, which increase their likelihood of developing the condition. Resistance exercise and nutritional support, which enhance muscle mass and strength, are key lifestyle strategies that can prevent or even reverse sarcopenia [42]. Exercise programs that combine resistance training with endurance exercises have been shown to significantly improve muscle function and strength [43]. Nutritional interventions, including adequate protein intake, vitamin D, and branched-chain amino acids, are considered beneficial for maintaining muscle mass [44]. However, although increased protein intake has been shown to stimulate muscle protein synthesis in the short term, evidence for its impact on muscle function is mixed and may vary across populations [44]. Emerging pharmacological treatments, such as myostatin inhibitors [45] have shown promising results in preclinical models [46]. However, clinical trials in humans have reported mixed results, with some improvements in muscle mass but inconsistent effects on muscle strength and functional outcomes [47]. These findings underscore the importance of raising awareness and systematically identifying individuals at risk for sarcopenia, allowing for timely interventions to help preserve muscle health and functional independence in older adults [45].

This study has several limitations. First, its cross-sectional design limits the ability to establish causal relationships between identified risk factors and the risk of sarcopenia. Second, the reliance on self-reported data for anthropometric measurements and health conditions may introduce recall or reporting bias. To minimize reporting bias, several strategies were implemented based on existing literature [48, 49]. These included providing ongoing training for our interviewers, using clear and straightforward questions, and conducting pilot testing before distributing the survey more widely. If participants encountered any difficulties in understanding a question, interviewers were available to offer explanations, ensuring that everyone understood that all data collected would remain anonymous.

Third, although the MSRA-5 is a practical and efficient screening tool for assessing sarcopenia risk, its diagnostic accuracy may vary [10, 16]. It does not directly measure muscle mass or strength, which are key components in diagnosing sarcopenia, highlighting the need for complementary clinical assessments. Despite these limitations, this study has notable strengths. In Israel, about 96% of individuals aged 65 and older reside in community settings. This study utilizes data from a nationally representative survey, enhancing its generalizability to the broader Israeli population of community-dwelling older adults. The inclusion of participants from diverse demographic and geographic backgrounds strengthens the study’s external validity. Additionally, this study contributes valuable insights into sociodemographic disparities and modifiable risk factors for sarcopenia, which can support the development of targeted prevention strategies.

## Conclusion

Our study highlights the high prevalence of sarcopenia risk among older adults in Israel and identifies key demographic and health-related factors associated with increased risk. Older age, Arab ethnicity, lower education levels, functional limitations, physical inactivity, polypharmacy, and chronic conditions such as heart failure and anxiety/depression were significant predictors of sarcopenia risk. These findings underscore the urgent need for targeted interventions, including physical activity promotion, medication review, and mental health support, to mitigate sarcopenia risk and improve health outcomes in aging populations. Given the growing aging population, early identification of individuals at risk is essential to enable timely interventions and prevent sarcopenia-related complications. Implementing routine screening for sarcopenia risk in clinical and community settings can facilitate early detection and intervention, ultimately helping to preserve mobility, independence, and overall quality of life among older adults.

## Data Availability

The data that support the findings of this study are available from the corresponding author, [DF], upon reasonable request.

## Abbreviations

BMI: Body mass index
CI: Confidence intervals
IADL: Instrumental Activities of Daily Living
INEF: Israel National Elderly Falls
MOH: Ministry of Health
MSRA: Mini Sarcopenia Risk Assessment
SARC-F: Strength, Assistance in walking, Rise from a chair, Climb stairs, and Falls questionnaire
SD: Standard deviation
